# Tribological Investigation of Textured Surfaces in Starved Lubrication Conditions

**DOI:** 10.3390/ma15238445

**Published:** 2022-11-27

**Authors:** Shubrajit Bhaumik, Viorel Paleu, Dhrubajyoti Chowdhury, Adarsh Batham, Udit Sehgal, Basudev Bhattacharya, Chiradeep Ghosh, Shubhabrata Datta

**Affiliations:** 1 Tribology and Interactive Surface Research Laboratory (TRISUL), Department of Mechanical Engineering, Amrita School of Engineering, Amrita Vishwa Vidyapeetham, Chennai 601103, India; 2 Mechanical Engineering, Mechatronics and Robotics Department, Mechanical Engineering Faculty, “Gheorghe Asachi” Technical University of Iași, 63 D. Mangeron Blvd., 700050 Iași, Romania; 3 Department of Mechanical Engineering, SRM Institute of Science and Technology, Kattankulathur, Chennai 603203, India; 4 Research and Development and Scientific Services, Tata Steel Limited, Jamshedpur 831001, India

**Keywords:** materials, friction reduction, micro-surface textures, vertical milling machine, artificial neural networks, optimal design

## Abstract

The present work investigates the friction reduction capability of two types of micro-textures (grooves and dimples) created on steel surfaces using a vertical milling machine. The wear studies were conducted using a pin-on-disc tribometer, with the results indicating a better friction reduction capacity in the case of the dimple texture as compared to the grooved texture. The microscopic images of the pin surface revealed deep furrows and significant damage on the pin surfaces of the groove-textured disc. An optimization of the textured surfaces was performed using an artificial neural network (ANN) model, predicting the influence of the surface texture as a function of the load, depth of cut and distance between the micro-textures.

## 1. Introduction

Friction is energy-consuming and is an important feature which all industries aim to control [1]. In spite of the use of lubricants [2] and surface modifications [3], surface texturing has emerged as a popular choice among many industries as a method to reduce friction. The methods for creating surface textures include photolithography [4], the laser method [5], wire-cut EDM, the ultrasonic method, grinding, chemical etching [6], etc. Surface textures are common on bearings [7,8], rings and seals [9,10,11], and implants [12]. The dimple texture is one such surface texture which has shown a significant decrease in the friction in the context of various lubrication regimes when manufactured using lasers [13,14,15,16]. Bhaduri et al. [17] tested laser-machined micro-dimple-textured surfaces with a ball-on-disc reciprocating tribometer and reported the third-body entrapment capability of the dimples. Vlădescu et al. [18] reported a 70% reduction in the coefficient of friction (COF) and an increased fluid film thickness with the use of the textured surfaces. Additionally, it was observed that with the increase in the number of dimples, the frictional properties were improved. Wakuda et al. [19] investigated the relationship between steel and ceramic surfaces in terms of the distribution of the micro-dimples and its effect on the friction. It was reported that the micro-dimples were not damaged under severe friction conditions, with the authors concluding that a proper morphology coupled with proper lubrication can succeed in preventing the appearance of harmful tribochemical layers. Li et al. [20] reported that high-density surface textures (13%) formed by laser peening are prone to friction reduction. Schneider et al. [21] reported that the use of hexagonal arrays of dimples (10% texture density and 0.2 aspect ratio) leads to an approximately 80% friction reduction for a mixed-lubricated regime using PAO oil.

Groove surface texturing is another type of texturing which involves the creation of a continuous path of textures, which are created using appropriate texturing tools. Wu et al. [22] investigated the importance of groove surface texturing in terms of the energy consumption and tribological properties. The grooves were fabricated using a laser on a 316 stainless steel specimen. The textures were of different widths and spacings but had the same depth and surface texture density of 35%. The tribometer was set in the oscillation mode under dry conditions. The authors concluded that the extent of the reduction in the friction is different under distinctive experimental conditions. Magri et al. [23] studied the application of surface texturing to forging dies and reported that the micro-cavities produced exhibited the best tribological properties due to the lubricant reservoir property of the cavities, leading to reduced adhesion between the tribo-pairs. Shuwen et al. [24] studied three different surface textures in the form of elliptical dimples, circular dimples and grooves using the laser surface texturing technique and reported the excellent performance of the friction and wear reduction (elliptical and circular), along with the stabilization of the friction in regard to the grooves’ texture. Duarte et al. [25] used the laser surface texturing methodology to produce groove patterns on commercially available stainless-steel samples. They observed that the textured surfaces produced performed well under starved lubricated conditions. The textures’ robust quality increased the lifetime of the lubricant film even under mixed lubricated conditions. Zhang et al. [26] studied the adhesion component of friction on human skin and the effects of the groove depth and sliding direction on the COF. It was observed that the groove textures were well suited for moderate values of the load when the direction of the sliding was taken into consideration. Groove channels manufactured using lasers have also been applied to ceramic surfaces. The geometrical shapes of the textures play an important role in the wear and COF of ceramic materials [27,28]. The tribological properties of dimple surface texturing and groove channels have been subjected to comparison. It should be noted that the friction reduction also depends on the shape, orientations and geometric dimensions of the textures [29,30,31,32,33]. Hu et al. [34] created dimples on 2024 aluminum alloy using laser texturing and reported a friction reduction with a dimple density of 8.5%. The reported results indicated that surface texturing can be used to control the friction. However, the cost requirements in the form of skilled labor and the availability of a high-end texturing process have always been concerns in the manufacturing industries. Though surface textures have been used for reducing the frictional properties, several researchers have also reported on the possible errors in the readings generated during the experiments and the ways to reduce such errors. Errors can also occur due to the incorrect calibration of the machines. It is therefore imperative that the readings be taken with the utmost care. Podulka [35] reported that the errors acquired during measurements and data processing can be reduced by thresholding methods that support the algorithms and the procedures followed. The usage of various filters, such as Gaussian, fast Fourier transform (FFT) and spline filters, can reduce the noise occurring in the readings. Heitjema [36] also indicated the possible problems that can arise when taking the readings. However, the author recommended the usage of the ISO 25178 standards to minimize the uncertainties occurring during the data recording.

The present work focusses on creating textures in the form of channels and investigating their tribological properties through an economical method, using a vertical milling machine which is available in any workshop of the industry. The results were compared with the authors’ previous work on dimples [37], which was also performed using a vertical milling machine. The focus of this work is the usage of the micro-surface textures formed using commonly available equipment, the vertical milling machine, which can help to minimize the process costs and, ultimately, reduce the end product cost. The comparison of the tribological behavior of the two different types of textures presented in this work and the manufacturing of the surface textures using the vertical milling machine can serve as a guideway for all manufacturing industries in order to curtail their costs of production when manufacturing surface textures. Moreover, this paper proposes the use of ANNs to improve the surface textures, assisting the design engineers in selecting the most effective solution. To the best of the authors’ knowledge, very limited work has been reported with respect to surfaces textures formed using vertical milling machines and the usage of ANNs for parametric investigations.

## 2. Materials and Methods

### 2.1. Materials

**Figure 1:**
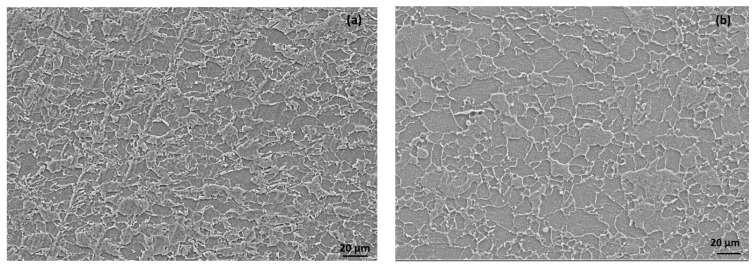
Micro-structure of (**a**) disc and (**b**) pin.

Two different types of low-carbon steels were used for the disc (0.075 % C, 1.49 % Mn, 0.033 % Si, 0.007 % S and 0.028 % P) and the pins (0.081 %C, 0.83 %Mn, 0.043 %Si, 0.006 %S and 0.025% P). The hardness of the disc was 250 BHN, and that of the pin was 157 BHN. The composition of the steels indicated the presence of almost equal amounts of carbon; however, the hardness differed due to the presence of manganese. The higher manganese (Mn) content in the steel of the disc resulted in a higher hardness. A microstructure analysis of the steels indicated the finer grain size of the disc as compared to the pin (Figure 1).

### 2.2. Texturing of the Surfaces (Groove Channels) Using a Vertical Milling Operation 

**Figure 2:**
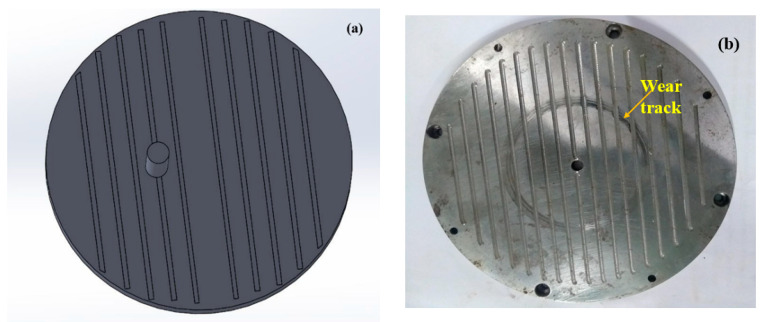
(**a**) Schematic arrangement of the pin on the perpendicular-groove-textured disc, and (**b**) wear track obtained on the textured disc.

**Figure 3:**
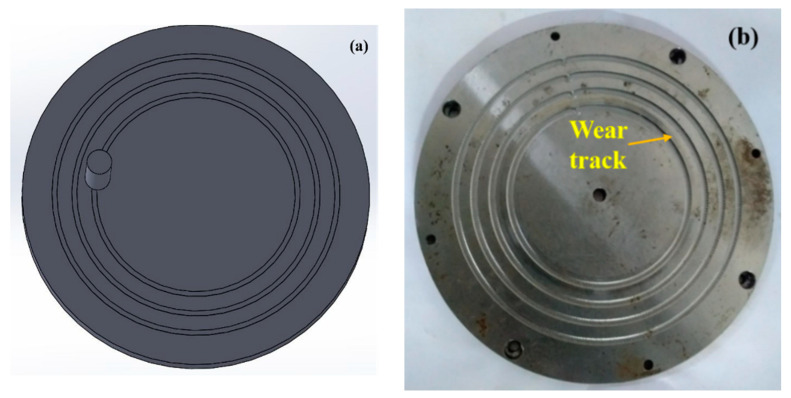
(**a**) Schematic arrangement of the pin on the circular-groove-textured disc, and (**b**) wear track obtained during experimentation.

A steel disc (Φ170 mm and 8 mm thickness) was used. The textures were created using a CNC machine (make: BFW; model: Gaurav BMV 35 T12). The milling tool was placed such that the tool was maintained as perpendicular to the surface to be machined. Grooves in the form of channels were created using the Sandvik R216F-0824 EL texturing tool, which is a cemented carbide ball-nose tool with an 8 mm diameter [23]. The tool was inserted into the CNC vertical milling machine, with a feed of 4221 mm/min and the tool rotating with 3500 rpm. The channels were formed such that the set of channels were perpendicular to the motion of the pin (Figure 2), termed as perpendicular grooves in this work, and the other set were along the motion of the pin, termed as circular grooves in this work (Figure 3). The material build-ups (if any) formed on the sides of the grooves need to be removed by soft grinding. All the material build-ups were carefully ground using a pencil grinder before using the disc for the tribo-tests. The two different orientations of the grooves were chosen for the following reasons:

i. The circular grooves act as a continuous reservoir of the lubricant and have continuous contact with the pin, while the perpendicular grooves have less contact with the pin.
ii. The lubricant confinement area is also different for both the grooves. Hence, under the centrifugal force due to the rotating disc, the amount of the lubricant that is present is not equal for each groove.
iii. It is easier to create circular and the perpendicular grooves, as no skilled labor is required by the industry. Hence, the cost of production can be reduced.

Furthermore, the choice of the friction couple, i.e., flat-on-flat, was chosen keeping in mind aspects of the applications, such as the side liners and gear tooth flank, where more sliding occurs, hence resulting in surface-to-surface contact.

### 2.3. Investigating the Tribological Properties of the Textured Surfaces and Surface Characterization

**Figure 4:**
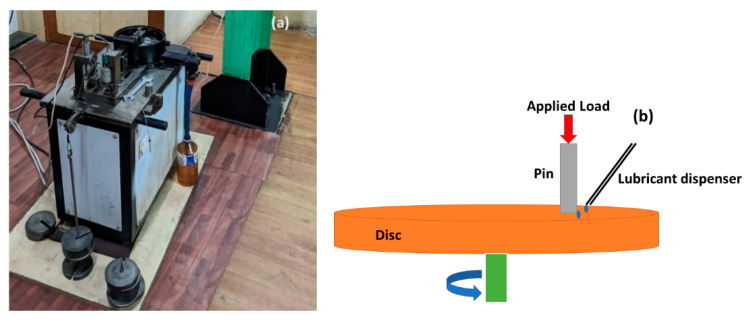
(**a**) Pin-on-disc equipment with lubrication facility, and (**b**) schematic diagram of the pin on the disc.

The authors reported that they were able to reduce the friction significantly by inscribing micro-dimples (a depth of 0.6 mm, diameter of 1.5 mm and spacing of 10 mm between them) [37]. However, during the process, it was also found that the depth of the dimples were difficult to control due to the material build-up, which caused the authors to consider a different set of textures, keeping in mind the use of the most economical method. A pin-on-disc tribometer (make: Magnum, model: POD1) was used to measure the tribological properties of the textured steel disc surface (Figure 4). The usage of a rotary tribometer was chosen in order to understand the efficiency of the textured surfaces in the presence of centrifugal force due to the rotation of the disc and the starved lubrication regime [37,38]. Cylindrical flat-faced steel pins (Φ10 mm and 30 mm length) were taken as the counterpart to the disc. The duration of each test was 3600 s. SAE 10W 30 oil was used as the lubricant, which was applied between the interface and the pin in a dropwise manner at a flow rate of 1 mL/s. This created a situation of insufficient lubrication between the mating pairs. Since there is a rotation motion in this procedure, the centrifugal force of the rotating disc will also disturb the flow of the lubricant, necessitating the condition of a minimal lubricant supply (lubrication starvation or starved lubrication). The surfaces of the pins were polished unidirectionally using emery papers of grit sizes of 400, 600, 800 and 1000. The surface roughness of all the pins were between 0.15 µm and 0.3 µm. The wear tests were performed with three types of textured discs: circular grooves in the direction of the motion of the pin, grooves perpendicular to the motion of pin and optimized dimples. The running parameters (load, speed of the disc, distance between the textures and depth of the cut) adopted in the experiments are shown in Table 1. In the case of the circular grooves, about 80% of the pin surface was on top of the groove; however, only one groove interacted with the pin surface. Meanwhile, in the case of the perpendicular grooves, almost all the grooves were in contact with the pin surface during the tribo-test. The frictional properties depend on the depth of the textures (as this is related to the formation of the hydrodynamic lift) and the texture density [32]. Hence, the depth of the cut and distance between the textures were chosen as the factors for controlling the friction.

An experimental table, as shown (Table 1), was followed to collect the experimental results for the values of the COF, which were later used as the dataset to develop the artificial neural network models (ANN). The experiments were limited due a shortage of materials, particularly the disc. Hence, the ANN analysis was employed to predict the results on the friction coefficient and wear over an extended range of input parameters. Additionally, to understand the interactions between the parameters, ANN models were developed together with the dataset. In order to comprehend the severity of the wear, the surfaces of the pins were analyzed using an optical microscope (make: Olympus).

### 2.4. Tolerances and Errors

The grooves were inscribed using a cemented carbide ball-nose tool with the diameter of 8.0 mm ± 0.01. These grooves were created using a CNC vertical milling machine of class 7 tolerance. Almost negligible material build-ups were seen along the grooves; however, if any build-ups were found, they were machined using a pencil grinder. The surfaces of the disc were free from any surface defects, and a roughness of 0.9 ± 0.01 µm to 1.01 ± 0.01 µm was maintained throughout. During the tests, a pin-on-disc tribometer was used, whose accuracy was 0.01, according to the manufacturer.

All the other machining was performed with the automatic CNC machine, with the manufacturing errors all being below 10 microns.

### 2.5. Implementation of Artificial Intelligence and Sensitivity Analysis to Investigate the Parametric Influence on the Frictional Coefficient

**Figure 5:**
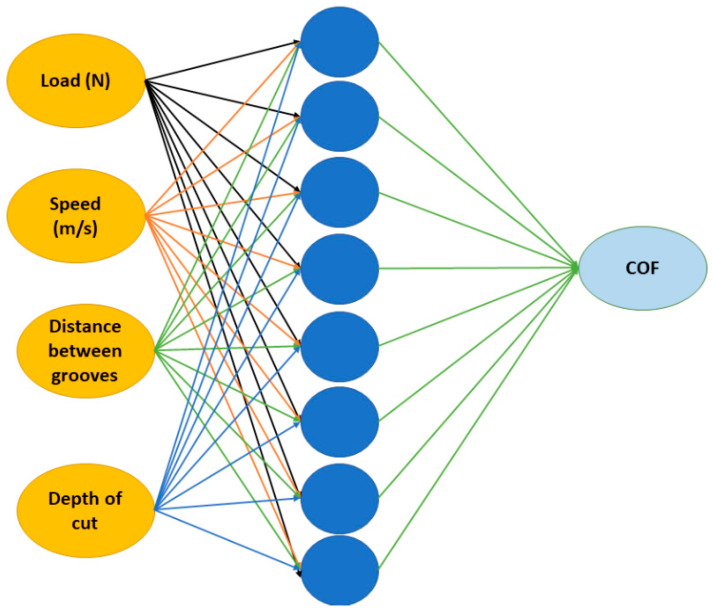
Typical 8-node ANN model used in this work.

Artificial intelligence helps us to understand the interactions between different parameters in critical conditions where the number of experiments are restricted due to material shortages or limited experimental conditions. Since the number of experiments was limited, artificial neural network (ANN) models were employed to predict the results over an extended input range. The input parameters for the ANN models were the data collected, as shown in Table 2. The ANN models were trained using the data generated during the experiments. The predicted output was the coefficient of friction. Similar to human synapses, ANN models also contain several neurons. A typical ANN model contains an input layer, hidden layer and output layer (Figure 5). A supervised multi-layered feed-forward network with a scale conjugate back-propagation algorithm was used in this work [38,39]. Several ANN models were generated using the data, and the model with highest regression coefficient was chosen for the present work. In this work, the highest regression coefficient was exhibited by an eight-node ANN model. The weighted inputs were normalized using Equation (1):

(1) $$A_{J}=tanh\left(\sum z_{ij}y_{i}^{N}+m_{j}\right),$$

where $A_{J}$ is the transfer function, and $z_{ij}$, $y_{i}$, $m_{j}$ are the weights and biases that govern the output prediction $A_{J}$.

The weights and biases of the output layer are taken from the hidden layer, and the linear weighted sum is calculated. The output OT is given by Equation (2):

(2) $$OT=\sum H_{j}A_{j}+b,$$

$\mathrm{where}\;H_{j}$ and *b* are the new biases. The predicted output is compared with the actual output and aims to reduce the error or the deviation.

Sensitivity analysis was conducted on the trained ANN models and was used to identify the set of influential parameters. Though several sensitivity methods are available [40], the present work used the connection weight method, which employs the hidden input and hidden output connection weights to calculate the importance of the variable [40]. The plots of the sensitivity were scaled along the *y*-axis from the minimum to the maximum on the COF.

## 3. Results and Discussions

### 3.1. Results of the Tribological Tests of the Perpendicular- and Circular-Grooved Surfaces

**Figure 6:**
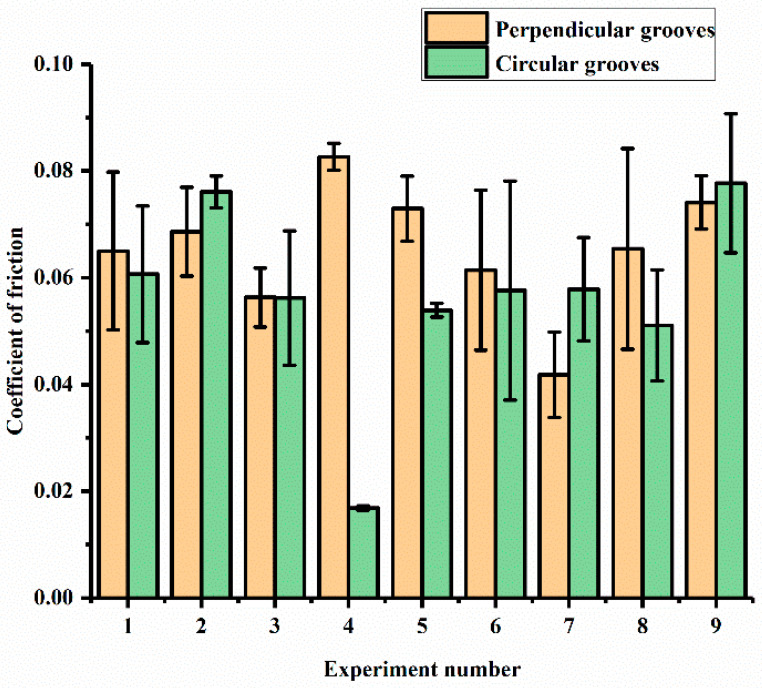
COF for perpendicular and circular grooves.

The tribological tests were conducted according to Table 1. In this set of experiments, the channels were manufactured in such a way that they were perpendicular to and in the direction of the motion of the pin, as indicated in Figure 2b and Figure 3b. Figure 6 shows the COF results obtained for the perpendicular and circular groove channels under the experimental conditions indicated in Table 1 for various loads (L), speeds (S), groove distances (D) and depths of cut (DP). For the perpendicular grooves, it can be seen that the combination of the factors in Experiment 4 (L:120N; S: 6.23 m/s; D: 4 mm; DP: 0.2 mm) exhibited the highest COF, while combination 7 (L:140N; S: 9.79 m/s; D: 2 mm; DP: 0.05 mm) exhibited lowest COF (almost 49% less). Experiment 5 and Experiment 9 also exhibited high COF values (0.085—0.075). Experiments 2, 6 and 8 showed moderate values of the COF in the range of 0.065—0.070. A slight increase in the value of the coefficient was observed in Experiments 1 and 2, where increases in the speed of the disc, distance between the textures and depths of the cut occurred, while the load remained constant. On further increases in the distance between the textures, speed of the disc and depth of the cut, a decrease in the COF occurred (Experiment 3). A sharp increase in the COF occurred in Experiment 4, where load was increased to 120 N, with a speed of 6.23 m/s, distance between the textures of 4 mm and a 0.2 mm depth of cut.

In the case of the circular grooves, it can be seen that Experiment 4 exhibited the lowest COF, while Experiments 2 and 9 displayed the highest values of the COF, which was in the range of 0.080—0.075 with a difference of 78.31% between the highest and the lowest values. Experiments 1, 3, 5, 6, 7 and 8 displayed similar values in the range of 0.06—0.05. A sharp rise in the value of the COF was observed in Experiment 2, where there was an increase in the speed of the disc, distance between the textures and depth of the cut. On further increases in the speed of the disc, distance between the textures and depth of the cut, the value of the COF in Experiment 3 remained similar to that of Experiment 1. A decrease in the value of the COF occurred in Experiment 4, where the applied load was increased to 120 N, the speed of the disc was decreased to 6.23 m/s, and 4 mm and 0.2 mm were the values of the distance between the textures and depth of the cut, respectively. This decrease may be due to the low disc speed, as the effect of centrifugal force would have been comparatively less significant in this experiment. Hence, the lubricant was able to maintain a stable film between the mating pairs. This result strongly indicates that it is important to consider the speed of the system when introducing texture into a tribo-system [41].

In Experiments 4, 5, 7 and 8, the values of the COF showed little variation in spite of the change in the values of the factors in various combinations. Experiment 9, however, showed a sharp rise in the value of the COF, obtaining the highest value obtained in this set of experiments. 

On comparing both types of groove textures, it was seen that Experiment 4 exhibited the lowest COF, whereas combinations 2 and 9 exhibited the highest COF values for the circular grooves. However, in the case of the perpendicular grooves, Experiment 4 exhibited the highest COF, whereas Experiment 7 exhibited the lowest COF. Additionally, the COF values for both the grooves were identical in Experiment 3. Overall, the value of the COF shown in Experiment 4 was lowest among all the displayed values for both textures in the different combinations. In Experiments 1, 4, 5, 6 and 8, the value of COF was less for the circular grooves than the perpendicular grooves, whereas the rest of the experiments showed high values of the COF for the circular grooves compared to the perpendicular grooves. From the above discussion, it can be seen that the shape and arrangement of the grooves can control the friction. However, because it was difficult for the authors to investigate the interactions between the parameters, the ANN models were used to understand these complex interactions.

### 3.2. Analysing the Effects of the Experimental Parameters on the Friction Coefficient Using an Artificial Neural Network

**Figure 7:**
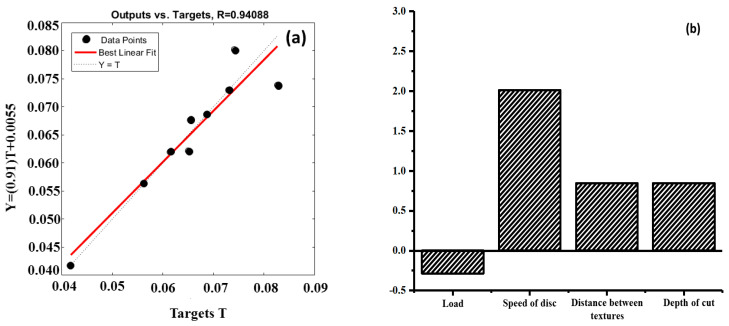

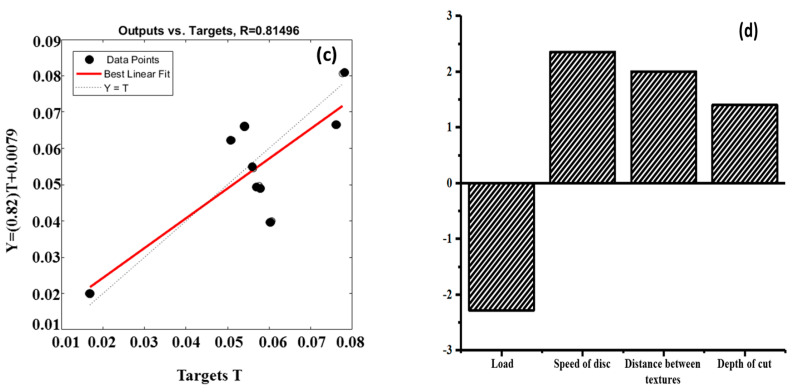
(**a**) Scatter plot for the circular grooves; (**b**) sensitivity analysis of the chosen ANN model for the circular grooves; (**c**) scatter plot for the perpendicular grooves; (**d**) sensitivity analysis of the chosen ANN model for the perpendicular grooves.

**Figure 8:**
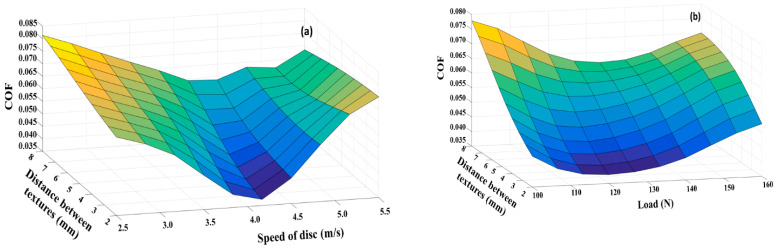

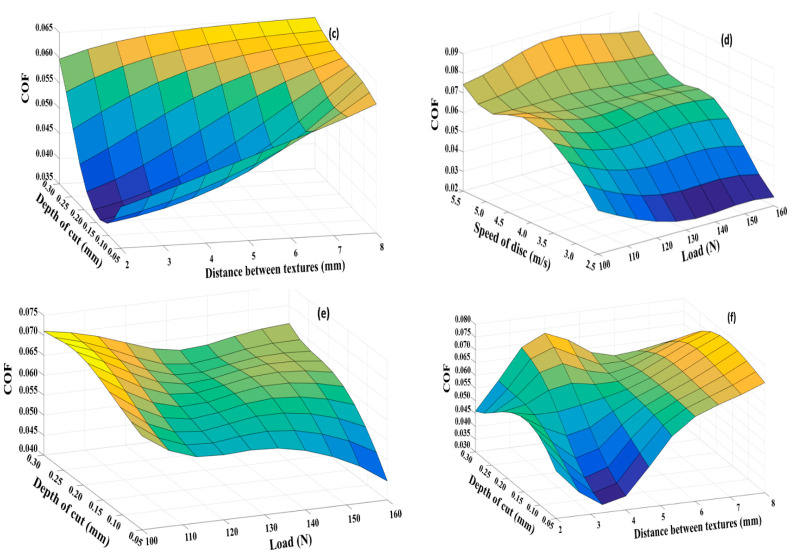
Surface interaction plots: (**a**–**c**) circular grooves; (**d**–**f**) perpendicular grooves.

The artificial neural network (ANN) model had four inputs (load, speed of the disc, distance between the textures, and depth of the cut), with one hidden layer and an output (COF). Before selecting the final ANN model, a number of ANN models were developed. However, the model with the highest regression coefficient was chosen, as shown in Figure 7a,c. The scatter plots showed high regression coefficients (R): 0.94088 for the circular grooves and 0.81496 for the perpendicular grooves, indicating a high experiment confidence level. The developed ANN models helped us to understand how the input parameters controlled the output parameter. The sensitivity analysis, as shown in Figure 7b,d, for both types of textures (circular and perpendicular) primarily indicated that with an increase in the speed of disc, distance between the textures and depth of the cut, there will be an increase in the COF values. The load seemed to have a negative effect in both cases, indicating a minimal effect on the COF. This is because with the increase in the load, the gap between the two mating pairs is reduced, hence causing more convergence at the ends of the mating pairs, providing better lubrication conditions. In order to gain a better understanding of the parameter interactions, surface plots were generated, as shown in Figure 8.

The surface plots (Figure 7) of the circular grooves with the applied ANN show that with the increase in the distance between the textures, depth of the cut and the speed of the disc, there was an increase in the COF. Additionally, the COF increased in the case of the circular grooves with the increase in the load. As seen from Figure 8a, the coefficient of friction initially decreased with the increase in the speed, but as the speed increased, the COF also increased. Similarly, as the distance between the grooves increased, the COF also increased (Figure 8b). Additionally, the increase in the depth of the cut (Figure 8c) increased the COF, indicating that shallow cuts perform better than deep cuts. This indicated that the hydrodynamic lift, in the case of the circular grooves, was not particularly effective. The non-confinement of the lubricant in the groove is the main reason, which resulted in the low upthrust from the lubricant in the grooves, which acted as reservoirs on the mating body (the pin). As the lubricant reservoirs were less effective, the lubricant film breakage was highly predominant and, hence, the COF also increased.

Though the results of the surface interactions of the perpendicular grooves were similar to those of the circular grooves, where the COF increased with the speed of the disc, depth of the cut and the distance between the textures (Figure 8d–f), the load seemed to show a positive effect in reducing the COF. This reduction is due to the greater confinement of the lubricant in the perpendicular grooves as compared to the circular grooves.

### 3.3. Comparison between the Groove Surface Textures and Dimple Surface Texture

**Figure 9:**
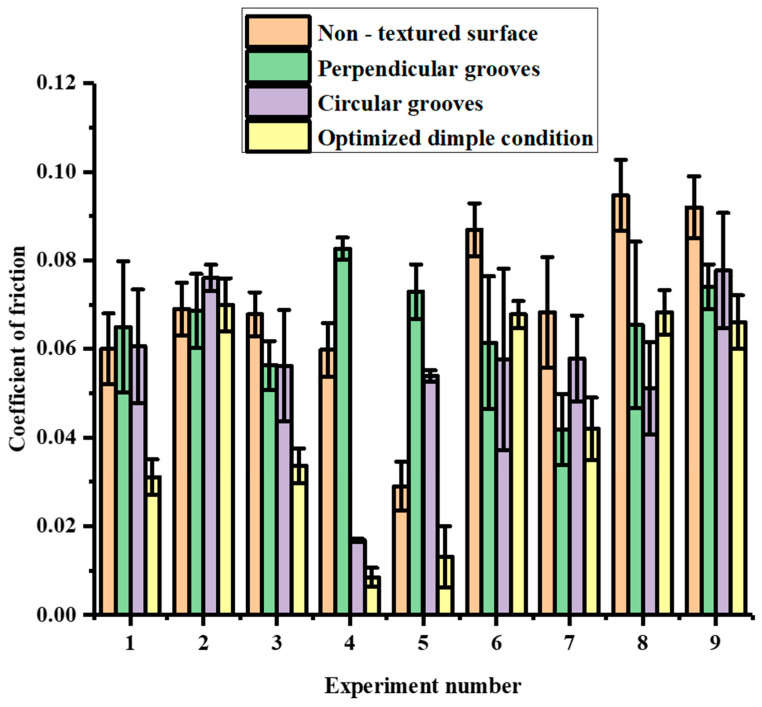
Comparison of the COF values as determined by different types of textures, along with the non-textured surface.

In the authors’ earlier work [37], it was observed that dimples with a geometry consisting of a 1.5 mm diameter, 0.6 mm depth and a spacing of 10 mm showed the optimal results. The same geometrical parameters were used to create the dimple textures in the present work. Figure 9 shows the comparison of the COF values as obtained in the experimentation, according to Table 1. In the case of the dimples and non-textured disc surface, only the load and speed of the disc were the varying factors in each of the experiments, as specified in Table 1.

From Figure 9, it was seen that optimized dimple geometry provided the lowest value of the COF with the combination of a 120 N load and speed of rotation of the disc of 6.23 m/s. The highest value was exhibited under the loads of 140 N and 160 N, with the speed of the disc being 6.23 m/s in both tests. The perpendicular groove channels performed best with the combination of a load of 140 N, when the speed of the disc was maintained at 9.79 m/s. Under a 120 N load and speed of the disc of 6.23 m/s, it exhibited highest value of the COF. The circular groove channels performed best with the combination of a load of 120 N and a speed of the disc of 6.23 m/s. Under a 100 N load and speed of the disc of 9.79 m/s, it displayed the highest value of the COF. The blank surface showed high values of the COF for almost all the combinations. 

Overall, with the disc bearing dimples, under a 120 N load and at the speed of the disc of 6.23 m/s, we obtained the lowest COF among all the textures. The perpendicular grooves that showed low COFs performed best in Experiments 2 and 7; however, Experiment 7 showed that the perpendicular channels had a similar COF value as compared to the dimpled surface with optimized geometrical parameters. The circular grooves exhibited the lowest frictional coefficient in Experiments 6 and 8. It should be noted that, except for Experiments 2, 6 and 8, the texturing in the form of dimples with optimized geometrical parameters exhibited the lowest frictional coefficient among all the samples. The dimple-textured surfaces exhibited an approximately 30%–40% reduced COF compared to the COFs of the textured and the non-textured surfaces. Furthermore, careful observation also indicated that the optimized dimple geometry reported by the authors in their earlier work exhibited the lowest COF in Experiment 4, indicating that the experimental parameters also played a major role in controlling the COF, particularly the speed of the disc. The lower speed enabled the lubricant to form a stable film due to the lower degree of centrifugal force, which, in other experiments, resulted in the lubricant moving away from the mating zone. The pin surfaces were further analyzed using the optical microscope to understand the severity of the wear under the influences of the different surface textures.

### 3.4. Analysis of the Wear Modes of the Pin Surface

**Figure 10:**
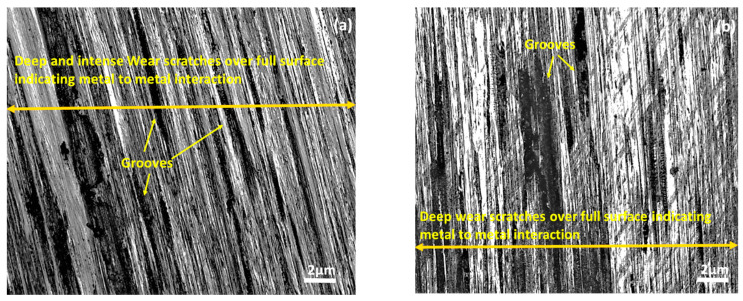

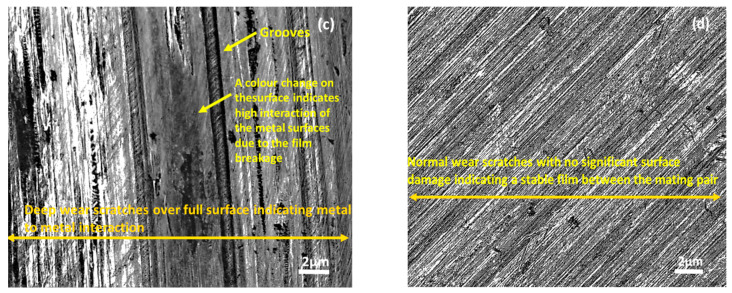
Optical microscope images of the pin at a magnification of 100× when moving against (**a**) non-textured disc, (**b**) circular grooves—when the pin slides in the direction of the groove, (**c**) perpendicular grooves—when the pin slides against the direction of the grooves, and (**d**) disc with the optimized dimple size.

As was seen earlier [42,43,44], the friction reduction capability of a textured surface depends on the size, shape and dimensions of the textures. The results obtained using different textures in the present work also depended on the types of the textures inscribed on the surface. On comparing the present results of the grooved textures with those of the dimpled texture, it was observed that the dimple-textured surfaces exhibited a better friction reduction capability than the groove-textured surfaces. The better performance of the dimple as compared to the channels indicated the presence of a better stable hydrodynamic lubrication regime in the case of the dimples. In addition to the relative velocity between the mating pairs, the inclination of the surfaces played an important role in creating a hydrodynamic regime [45]. In the case of a dimple, near to the converging end of the dimple, the lubricant will become more concentrated and, hence, will exert higher pressure on the counter body, thus forcing the pin upwards and separating it from the disc [46]. However, in the case of the channels, there was no confinement of the area and, hence, the lubricant in the channels was not able to generate sufficient hydrodynamic pressure. Nakano et al. [47] also indicated a better hydrodynamic regime in the case of dimples. The present results exhibited that the surfaces of the pins which came into contact with the disc bearing dimples experienced less damage as compared to the pin surfaces which came into contact with the non-textured discs and the discs bearing parallel grooves and circular grooves (Figure 10). The presence of several grooves on the pin surfaces which interacted with the untextured disc surface (Figure 10a), circular grooves (Figure 10b) and perpendicular grooves (Figure 10c) indicated the breakage of the lubricant film. Careful observations of the pin surface in the case of the perpendicular grooves indicated the presence of a color difference (Figure 10c) corresponding to close contact between the mating pairs. All these observations showed the high chance of lubricant film failure at several points during the tribo-tests. Thus, the pins of all the samples except for the one which came into contact with the disc bearing dimples experienced severe damage, proving the existence of the unstable lubricating film.

## 4. Conclusions

The purpose of this study was to explore the possibility of adopting micro-textures that are manufactured using less expensive methods, namely, vertical milling. Pin-on-disc tribometer tests were used to evaluate the effectiveness of textured surfaces with perpendicular, circular and dimpled grooves in reducing the wear and COF values under starved lubrication conditions. A relationship between the COF, speed, applied load and geometrical parameters of textured surfaces was predicted using the ANN method.

According to the obtained results, the following conclusions can be drawn:

1. Following the experimental results obtained from the tests using the pin-on-disc machine, regarding the minimum coefficients of the friction and wear, it was observed that the confined textures in form of dimples performed better than the grooved textures, which were not able to provide a confined area for the entrapped lubricant. The confined area promotes the presence of hydrodynamic conditions more effectively than the non-confined areas.
2. Extending the results of the experimental work with the aid of the ANN models, we proved that an increase in the speed of the disc, distance between the textures and depth of the cut will also lead to an increase in the coefficient of friction values. With the ANN, the optimization of the textured surfaces can be performed safely by taking into consideration not only the geometric parameters but also the running parameters, such as the speed and load.
3. According to the surface characterization of the tested samples, the groove-textured surfaces acquired deep ploughing traces, while the dimpled surfaces exhibited no damage. Hence, it was proven that the textures machined by the economical milling process were capable of reducing the friction and wear under starved lubricating conditions.
4. Taking all of the observations from this paper into account, it can be concluded that micro-textures created using milling machines can reduce friction (especially in the case of dimples) and, thus, reduce labor and production costs of processing industries. However, material build-up on the edges must be eliminated during the manufacturing process of micro-dimples.

Summarizing the observations in this work, it can be concluded that micro-textures created using milling machines can control friction (particularly the dimples) and, hence, this can result in reductions in the labor and production costs of process industries. However, the material build-up on the edges needs to be eliminated when manufacturing the micro-dimples.

Future work on the generation of complex micro-textures using vertical milling machines is planned, including the complex shapes of the textures, such as hybrid geometrical shapes comprising features from two or more geometries. The present work considered a single viscosity lubricant and, hence, the effect of viscosity on the frictional properties in the presence of textures can be explored in the upcoming research. Additionally, the present research was performed at room temperature; thus, the influence of the temperature on the tribological contact of the textured surfaces in dry friction and oil-starved lubrication conditions would also be an interesting topic of study.

## Figures and Tables

**Table 1:** Experimental planning.

Experiment Number	Load (N)	Speed of Disc (m/s)	Distance between Textures (mm)	Depth of Cut (mm)
1	100	8.01 ± 0.1	4 ± 0.01	0.1 ± 0.01
2	100	9.79 ± 0.1	6 ± 0.01	0.2 ± 0.01
3	100	11.57 ± 0.1	8 ± 0.01	0.3 ± 0.01
4	120	6.23 ± 0.1	4 ± 0.01	0.2 ± 0.01
5	120	11.57 ± 0.1	6 ± 0.01	0.1 ± 0.01
6	140	6.23 ± 0.1	6 ± 0.01	0.3 ± 0.01
7	140	9.79 ± 0.1	2 ± 0.01	0.05 ± 0.01
8	160	6.23 ± 0.1	8 ± 0.01	0.1 ± 0.01
9	160	9.79 ± 0.1	4 ± 0.01	0.3 ± 0.01

**Table 2:** Input parameters for the ANN models.

Input Variables	Minimum	Maximum	Average	Standard Deviation
Load (N)	100	160	127.27	24.49
Speed of disc (m/s)	6.23	11.57	8.81	2.20
Distance between textures (mm)	2	8	5.27	2
Depth of cut (mm)	0.05	3	0.18	0.1
